# Identification and characterization of novel Kirrel isoform during myogenesis

**DOI:** 10.1002/phy2.44

**Published:** 2013-08-22

**Authors:** Peter J Durcan, Nasser Al-Shanti, Claire E Stewart

**Affiliations:** 1 Department of Physiological Sciences, Stellenbosch UniversityMerriman avenue, Stellenbosch, 7600, Western Cape, South Africa; 2 Institute for Biomedical Research into Human movement, School of Healthcare Science, Manchester Metropolitan UniversityOxford road, M1 5GD, Manchester, U.K.; 3 Research Institute for Sport and Exercise Sciences, School of Sport and Exercise Sciences, Liverpool John Moores UniversityLiverpool, U.K.

**Keywords:** Cell fusion, drosophila, Kirrel, myogenesis

## Abstract

Somatic cell fusion is an essential component of skeletal muscle development and growth and repair from injury. Additional cell types such as trophoblasts and osteoclasts also require somatic cell fusion events to perform their physiological functions. Currently we have rudimentary knowledge on molecular mechanisms regulating somatic cell fusion events in mammals. We therefore investigated during in vitro murine myogenesis a mammalian homolog, Kirrel, of the *Drosophila Melanogaster* genes Roughest (Rst) and Kin of Irre (Kirre) which regulate somatic muscle cell fusion during embryonic development. Our results demonstrate the presence of a novel murine Kirrel isoform containing a truncated cytoplasmic domain which we term Kirrel B. Protein expression levels of Kirrel B are inverse to the occurrence of cell fusion events during in vitro myogenesis which is in stark contrast to the expression profile of Rst and Kirre during myogenesis in *Drosophila*. Furthermore, chemical inhibition of cell fusion confirmed the inverse expression pattern of Kirrel B protein levels in relation to cell fusion events. The discovery of a novel Kirrel B protein isoform during myogenesis highlights the need for more thorough investigation of the similarities and potential differences between fly and mammals with regards to the muscle cell fusion process.

## Introduction

Generation of the syncytial trophoblast during embryonic development, formation of syncytial skeletal muscle fibers, and development of osteoclasts highlight important developmental processes in mammals which are underpinned by somatic cell fusion events. The occurrence of somatic cell fusion has been reported in additional tissues including heart (Dedja et al. 2006), liver (Faggioli et al. 2008; Fujimiya et al. 2007), brain (Alvarez-Dolado et al. 2003; Johansson et al. 2008), prostate (Placencio et al. 2010), and the intestinal epithelium (Davies et al. 2009; Rizvi et al. 2006). Furthermore, it has been hypothesized that somatic cell fusion could be a mechanism enabling cancer metastasis and in generating resistance to chemotherapy treatments (Duelli and Lazebnik 2003; Pawelek and Chakraborty 2008). The diverse array of cell types affected by somatic cell fusion events demonstrates the need for intense study of this rudimentary understood process. In specific tissue types, such as skeletal muscle, thousands of cell fusion events can take place during the development of a single human muscle fiber in vivo (Peckham 2008); however, these fusion events are asynchronous and hence suggest that the somatic cell fusion process is under temporal control of specific genes.

Significant advances have been made in improving our understanding of how the somatic cell fusion process is regulated during embryonic development of the body wall musculature in the invertebrate model system*, Drosophila* melanogaster (Abmayr and Pavlath 2012). Research findings from *Drosophila* are increasingly utilized in diverse research disciplines to strengthen and develop hypotheses regarding the molecular underpinnings of development and diseases in humans (Botas 2007). Indeed, evidence is emerging which supports the concept of conservation of key signaling networks between *Drosophila* and mammals in regulating the somatic cell fusion process. The dedicator of cytokinesis (Dock) protein family member Dock1, which is an atypical guanine nucleotide exchange factor (GEF) for Rac1 has been demonstrated to be an important regulator of the in vivo muscle cell fusion process in *Drosophila* (Erickson et al. 1997) and mouse models (Laurin et al. 2008). In addition, an mRNA transcript for the Type-1 transmembrane protein Nephrin which is a member of the immunoglobulin superfamily has recently been reported to be present in human primary skeletal muscle cell cultures and in mouse skeletal muscle during the healing response to cardiotoxin-induced injury (Sohn et al. 2009). Nephrin is the mammalian homolog to the *Drosophila* gene sticks and stones (SnS) (Bour et al. 2000). Loss of SnS results in inhibition of the muscle cell fusion process during embryonic development in *Drosophila* (Bour et al. 2000), and intriguingly, loss of Nephrin in zebrafish and in mouse muscle cells in vitro results in decreased somatic cell fusion events (Sohn et al. 2009). During the occurrence of muscle cell fusion events in *Drosophila* SnS colocalizes in trans at the cell membrane of fusing muscle cells with an additional Type-1 transmembrane protein and member of the immunoglobulin superfamily, Kin of Irre (Kirre) (Galletta et al. 2004; Sens et al. 2010). Present within the *Drosophila* genome is a Kirre paralog termed Roughest (Rst) (Strünkelnberg et al. 2001). Elimination of both Kirre and Rst results in complete inhibition of the muscle cell fusion process (Strünkelnberg et al. 2001). The mammalian homologs to Rst and Kirre are the Kirrel gene family, Kirrel, Kirrel2, and Kirrel3 (Neumann-Haefelin et al. 2010). Currently we have rudimentary knowledge regarding the murine Kirrel family in skeletal muscle as the vast majority of research on this gene family has focused on its role in the slit diaphragm of the mammalian kidney (Donoviel et al. 2001; Gerke et al. 2003; Liu et al. 2003) or in brain development (Gerke et al. 2006; Nishida et al. 2011; Tamura et al. 2005). The *Caenorhabditis. elegans* homolog of the Kirrel family is synaptogenesis abnormal 1 (SYG-1) and it has been implicated in neural synapse formation (Shen and Bargmann 2003). We therefore wished to initially examine one of the Kirrel family members, Kirrel, during in vitro myogenesis to assess if evidence could be found which would support a possible role for Kirrel in regulating the somatic cell fusion process in murine skeletal muscle. Our results identify a previously unreported splice variant of Kirrel which is present in murine muscle cells during in vitro myogenesis and also in the mouse brain. Alternative splicing is predicted to lead to the production of a truncated protein compared to the previously reported Kirrel (Liu et al. 2003) which would result in significant alternations in the cytoplasmic domain of Kirrel. We termed this truncated Kirrel transcript Kirrel B. We also present evidence that expression levels of the Kirrel B protein isoform are surprisingly inverse to occurrence of somatic cell fusion events during in vitro myogenesis which is in stark contrast to the expression profile of Kirre and Rst during myogenesis in *Drosophila*, whereby their expression is highest when the greatest number of cell fusion events are occurring (Ruiz-Gómez et al. 2000; Strünkelnberg et al. 2001). Furthermore, to substantiate the inverse link between Kirrel B expression levels and somatic cell fusion events, we chemically inhibited the cell fusion process which resulted in significant upregulation of the Kirrel B protein isoform. Our results are particularly noteworthy in light of work on Kirrel homologs Rst and Kirre in *Drosophila* myogenesis (reviewed in Abmayr and Pavlath 2012 and additionally on the *C. elegans* homolog of Kirrel, SYG-1 in synaptogenesis [Shen and Bargmann 2003]). Further work is required to ascertain how Kirrel B may be involved in regulating diverse physiological processes such as muscle cell fusion and neurogenesis.

## Material and Methods

### Materials

C2C12 cells were obtained from ATCC. All plastic ware unless otherwise stated were obtained from Fischer scientific. Dulbecco's modified eagles medium (DMEM) was obtained from Lonza (Slough, U.K.). Heat-inactivated (HI) new born calf serum (NCS) and HI fetal bovine serum (FBS) were obtained from Gibco (Paisley, U.K.). HI horse serum (HS) was from Southern Group Laboratory (Corby, U.K.). l-glutamine was obtained from BDH (Poole, U.K.), and penicillin streptomycin solution and trypsin were obtained from Bio Whittaker (Wokingham, U.K.). Gelatin Type A from porcine skin primers for two-step reverse transcription polymerase chain reaction (RT-PCR) and all chemicals unless otherwise state were obtained from Sigma Aldrich. Phosphate-buffered saline was from Oxoid Ltd., (Basingstoke, U.K.). Bisperoxo(5-hydroxypyridine-2-carboxyl) oxovanadate (BpV) was obtained from Calbiochem (Darmstadt, Germany). Trizol, TaqMan® RNA-to-CT™ one-step kit, Taq man probes, DNA-free™ Dnase, nuclease-free water, and TE buffer pH 8.0 were all obtained from Life Technologies (Paisley, U.K.). PCR plates for qPCR were obtained from Bio-rad (Hercules, CA). UV plates for creatine kinase (CK) assay were obtained from BD Biosciences (Oxford, U.K.).

### Cell culture

C2C12 murine skeletal myoblast from ATCC (Blau et al. 1985) was initially grown in T75 flasks in a humidified 5% CO2 atmosphere at 37°C in growth medium (GM), composed of: DMEM plus 10% hi FBS, 10% hi NCS, 1% l-glutamine which was sterile filtered (2 mmol/L final), and 1% penicillin–streptomycin solution, until 80% confluence was attained. Experiments were subsequently initiated by trypsinization of adherent cells and seeding cells in GM at a density of 40 × 10^3^ cells/mL for 12-well plates (1 mL total volume) or 50 × 10^3^ cells/mL for 6-well plate (2 mL total volume). Plates had been prior coated with 0.2% gelatin for 5 minutes at room temperature with excess gelatin aspirated prior to cell seeding. Upon attaining 90–100% confluency GM was removed and cells were washed once with phosphate buffered saline (PBS). For the 0-h time point cells were subsequently lysed at this stage. For later differentiation, time points differentiation media (2% HS, 1% penicillin–streptomycin solution, and 2 mmol/L l-glutamine) were added (1 mL per well for 12-well plate, 2 mL per well for 6-well plate) for indicated time points and subsequently were removed with cells being washed one time with PBS prior to lysis with the desired lysis buffer (see below).

### Cell treatments and extractions

For both the BpV and nutrient challenge study, the 0-h time point described above was the initiation time point. Differentiation medium (DM) containing 10 μmol/L BpV (Bpv dissolved in distilled H_2_O) or DM without BpV was added to cells which were grown simultaneously in three independent experiments. For RNA isolation experiments, cells were grown in 12-well plates, and 150 μL of Tri Reagent (Ambion, U.K.) was added to each well and left at room temperature for 5 min with occasional agitation to enable cell lysis. For sodium dodecyl sulfate polyacrylamide gel electrophoresis (SDS-PAGE), cells were grown in 6-well plates and 150 μL of SDS-PAGE lysis buffer (10 mmol/L Tris-Cl, 5 mmol/L ethylenediaminetetraacetic acid, 50 mmol/L NaCl, 30 mmol/L Na_4_P_2_O_7_, 50 mmol/L NaF, 100 μmol/L Na_3_VO_4_, 1 mmol/L phenylmethanesulfonyl fluoride, and 1% Triton X-100 pH 8.3) was added per well. Cells were left on ice for 5 min to enable lysis with occasional agitation and subsequently cell scrapers were used to aid in complete lysate removal. All samples were stored at −80°C until analysis.

### Microscopy

For capture of phase contrast cell images, a cell imaging system at 10× magnification (Leica, DMI 6000 B, Wetzlar, Germany) was used.

### Animal tissue RNA and protein isolation

All mouse tissue was obtained from c57/BL6 mice. Whole-brain samples D16 (*n* = 6) were kindly provided by Dr. Stuart Lanham Southampton University. For RNA isolation, whole brains (*n* = 3) were pooled and homogenized on ice in 1 mL of Tri reagent. For RNA isolation from adult male Wistar rats (kind gift of Dr. May Azawazi Manchester Metropolitan University), 20 mg of tissue was obtained via 20-μm cryosections, placed in 1 mL of Tri reagent and subsequently homogenized on ice.

### Rt-pcr

Ribonucleic acid concentration was determined using a Biotech Photometer (WPA UV1101, Biochrom, Cambridge, U.K.). For two-step RT-PCR using custom-designed primers, 2 μg of RNA was DNase treated and reverse transcribed using the Quantitect reverse transcription kit (Qiagen, U.K.) according to manufactures guidelines. A 2-μL cDNA aliquot was subsequently used in 25-μL total volume PCR which was conducted in accordance with manufacturers (Taq Core Qiagen, Crawley, U.K.) guidelines on an Eppendorf master cycler (Eppendorf, U.K.). Annealing temperatures of primers were 55°C and primer concentration was 2.5 μmol/L. Primer sequences 5′-3′ used were as follows: **Kirrel A-specific primer set** – forward primer CGTGGAGAGGACGAACTCAG and reverse primer GGCACGGTAGTCAGCATACA; and **Kirrel B-specific primer set –** forward primer ATGAGAGTCGCTATGAGACAACG, reverse primer – GCCGTAGGACAATGAAGAGC. PCR products were run on a 1% agarose gel alongside a 100 bp ladder (Invitrogen, U.K.) for size quantification and visualized by ethidium bromide staining and UV detection on a Bio-rad Gel Doc™ XR supported by Quantity One 4.6.2 (Bio-rad).

For qPCR, RNA was DNase treated with DNA free (Life Technologies) according to manufacturer's guidelines, and subsequently, 160 ng of DNase-treated RNA was used per qPCR reaction (20 μL total volume). Each sample was run in duplicate on a 96-well plate (Bio-rad). TaqMan® RNA-to-CT™ one-step (Life Technologies) was used for qPCR. Thermal cycler (Chromo4™ DNA engine Biorad) conditions were used as recommended for TaqMan® RNA-to-CT™ one-step kit by manufacturer. TaqMan probe used for Kirrel was Mm01209463. Gene expression levels were calculated using the comparative 2^−ΔΔCT^ method (Livak and Schmittgen 2001), where RNA polymerase II DNA-directed polypeptide β (polr2β; NM_153798) was used as reference gene (TaqMan probe Mm00464214), as this gene has been previously validated by our research group (Dimchev et al. 2013; Sharples et al. 2010). For analysis of Kirrel gene expression during C2C12 differentiation, consecutive hours were grouped 20, 21, and 22 h (20–22 h); 40, 41, and 42 h (40–42 h); and 70 h, 71, and 72 h (70–72 h) to give an accurate indication of Kirrel expression at specific stages of myogenesis, that is, Days 1, 2, and 3 and are expressed relative to 0-h time point.

### SDS-PAGE and immunodetection

Protein concentration of cell lysates was determined using the bicinchoninic acid assay (Pierce, Thermo Fischer Scientific, U.K.) and Bovine serum albumin (BSA) concentration standards. Protein concentration was measured on a Bio Tek Elisa Plate reader EL×800 (Bedfordshire, U.K.). Forty μg of protein was loaded per lane for all samples with 1× Lamelli buffer (50 mmol/L Tris-HCL, pH 6.8, 10% glycerol, 2% SDS, 1% mercaptoethanol, and 0.1% bromophenol blue). Samples were heated at 100°C for 5 min and then spun (1 min 5000*g*). SDS-PAGE was performed with a 7% resolving gel using a pharmacia biotech power supply (EPS 3500) and Hoefer scientific gel casting system (SE600). Semidry transfer (BDH Semi-Dry electroblotter Merck Eurolab, Dorset, U.K.) was subsequently conducted onto nitrocellulose membranes (Amersham GE Healthcare, Buckinghamshire, U.K.). Equal protein loading and transfer was confirmed via Ponceau S staining. Membranes were subsequently washed to remove Ponceau S and then blocked with 5% semiskimmed milk in 1× tris buffered saline with tween (TBST) (50 mmol/L Tris, 150 mmol/L NaCl, and 0.1% Tween 20). Membranes were then incubated overnight at room temperature (18–20°C) with primary polyclonal rabbit anti-human Kirrel antibody (Abcam ab82804), 1:4000 dilution in 5% semiskimmed milk in 1× TBST with gentle agitation, and were subsequently washed 3× for 5 min and incubated with horse raddish peroxidase-conjugated goat antirabbit secondary antibody (Mp biomedical), 1:25,000 in 1× TBST with 5% semiskimmed milk for 1 h at room 18–20°C. Following secondary antibody incubation, membranes were washed 4× for 5 min in 1× TBST. Subsequently membranes were incubated with SuperSignal West Femto–enhanced chemiluminescence (ECL) reagents (Pierce, Thermo Fischer Scientific). Light intensity was captured via Chemi Doc™ XRS (Bio-rad) which was supported by Quantity one 4.6.2 software (Bio-rad). Postdetection of Kirrel, membranes were washed 2× with 1× TBST and reincubated for 4 h at 18–20°C with a monoclonal rabbit antimouse β-Actin (New England Biolabs -5125, Hertfordshire, U.K.) primary antibody, 1:5000 suspended in 1× TBST with 3% BSA. Detection of β-Actin was as described for Kirrel. For analysis of Kirrel protein expression during C2C12 differentiation, consecutive hours were grouped 20, 21, and 22 h (20–22 h); 40, 41, and 42 h (40–42 h); and 70, 71, and 72 h (70–72 h) to give an accurate indication of Kirrel expression at specific stages of myogenesis, that is, Days 1, 2, and 3.

### Statistics

Statistical analyses were carried out using SPSS version 18. One-way analysis of variance (ANOVAs) were carried out when >2 comparisons were being made and Bonferroni post hoc tests were subsequently carried out to obtain statistical significance. When only two comparisons were being made, student's *t*-test was used. Statistical significance was set at *P* < 0.05. Values are expressed as mean ± standard error of mean (SEM).

## Results

### Identification of Kirrel B

**Figure 1:**
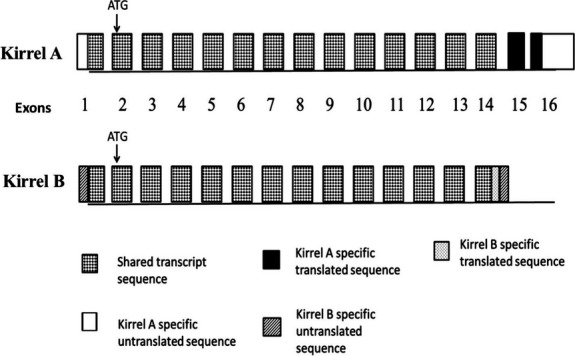
Schematic illustrating exon structure and transcript differences between Kirrel A and Kirrel B. ATG codon prediction obtained from open-read frame finder software available at (http://www.ncbi.nlm.nih.gov/projects/gorf).

Searching of the murine National Centre for Biotechnology Information (NCBI) database (http://www.ncbi.nlm.nih.gov) for Kirrel yielded two validated transcripts NM_001170985.1 (hereafter known as Kirrel A) and NM_130867.3, the former being three nucleotides longer at 7287 bases. The small discrepancy maps to a splice site at the 3′ end of the untranslated first exon; however, both transcripts encode the same mature Kirrel protein. The consensus coding sequence (CCDS) for murine Kirrel is CCDS17450.1. The Basic Local Assignment Search Tool (BLAST) available at http://blast.ncbi.nlm.nih.gov/blast.cgi was utilized to search the NCBI murine nonredundant nucleotide (nr/nt) database for possible previously unreported splice variants of Kirrel using the CCDS17450.1 nucleotide sequence as template. This search yielded a previously unreported mRNA sequence bc023765 (hereafter referred to as Kirrel B) which contained 2228 nucleotides. The Kirrel B mRNA transcript, which contains a poly A tail, was identified in a murine mammary tumor. Aligning the transcript sequences of Kirrel A and Kirrel B to the mouse genome via the genomic sequence present in NT_039240.7, it was found that Kirrel A and B contain 16 and 14 exons, respectively (see Fig. 1 for schematic). Kirrel A has an additional two unique exons (of 79 and 5030 nucleotides) at its 3′ end and an additional 197 nucleotides at its 5′ end which are not present in Kirrel B. Kirrel A and Kirrel B are predicted via the open-reading frame finder software (available at http://www.ncbi.nlm.nih.gov/projects/gorf/) to share the same ATG translation start codon in their second exons at nucleotide 366 and 179, respectively. Between nucleotides 11 and 1993 of Kirrel B, which spans exons 1–14 of both transcripts, identical sequence data are present in Kirrel A. The first 10 nucleotides of Kirrel B do not map to the murine genome and the reason for this is currently unknown. A missed spliced site that is present in exon 14 of Kirrel A at position 2180 results in the production of a truncated Kirrel B transcript with a thymine, Adenine, Adenine (TAA) stop codon present 87 nucleotides 3′ to the missed spliced site. Following the TAA stop codon, a 3′ untranslated region of ∼85 nucleotides is present prior to the poly A tail. Comparing the protein-coding sequence of CCDS 17450.1 with the Kirrel A transcript sequence it was observed that Kirrel A contains a significantly larger 3′ untranslated region of approximately 4550 nucleotides.

### *In silico* analysis of protein structures for Kirrel A and B

**Figure 2:**
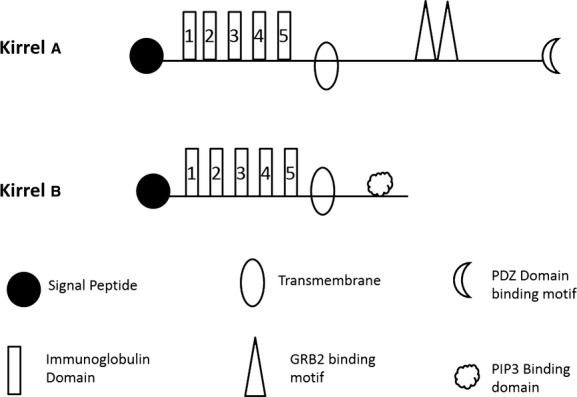
Schematic of a selection of structural domains present in Kirrel A and Kirrel B. Signal peptide, immunoglobulin domains, and transmembrane domain prediction obtained from UniProt (http://www.uniprot.org). Cytoplasmic structural domains obtained from preforming a high stringency scan via Scansite (http://scansite.mit.edu). Y638 was not predicted by Scansite, however, this site has previously been reported to be essential for Grb2 binding (Harita et al. 2008). PDZ domain in Kirrel was previously reported (Sellin et al. 2003).

The predicted molecular weights of unmodified Kirrel A (NP_001164456.1) and Kirrel B (AAH23765) isoforms are ∼87 kDa and 70 kDa, respectively. Kirrel A contains 789 amino acids (aa), whereas Kirrel B is shorter at 634 aa. The first 605 aa of both isoforms are identical. This homologous coding sequence (see Fig. 2 for schematic) encodes a region spanning from the signal peptide to the 53rd cytoplasmic amino acid. Domains present in this homologous sequence include a signal peptide aa 1–47 (http://www.cbs.dtu.dk/services/signalip/); five extracellular immunoglobulin domains (Ig): aa 54–151, 151–243, 256–339, 340–422, and 424–509 (http://scansite.mit.edu); and a transmembrane domain between aa 529 and 551 (http://www.cbs.dtu.dk/services/tmhmm/). The cytoplasmic domain of Kirrel A subsequently differs to that of Kirrel B as it contains two tyrosine (Y) residues (Y637 and Y638) which regulate growth factor receptor bound 2 (Grb2) binding in in vitro assays and also in Kirrel A pull down assays for Grb2 from rodent kidney lysates (Garg et al. 2007; Harita et al. 2008). A post synaptic density protein 95, *Drosophila* disc large tumour suppressor, zonula occludens 1 (PDZ) binding domain motif is also present at the c-terminus of Kirrel A aa 787–789 (Sellin et al. 2003). While the truncated cytoplasmic domain present in Kirrel B leads to the loss of the Grb2 and PDZ motifs, a putative phosphatidylinositol-3,4,5-triphosphate pleckstrin homology (PIP3 PH) motif is predicted between aa 607 and 621(http://scansite.mit.edu). This domain is not predicted to be present in Kirrel A.

### Expression profiling of Kirrel A and B mRNA transcripts

**Figure 3:**
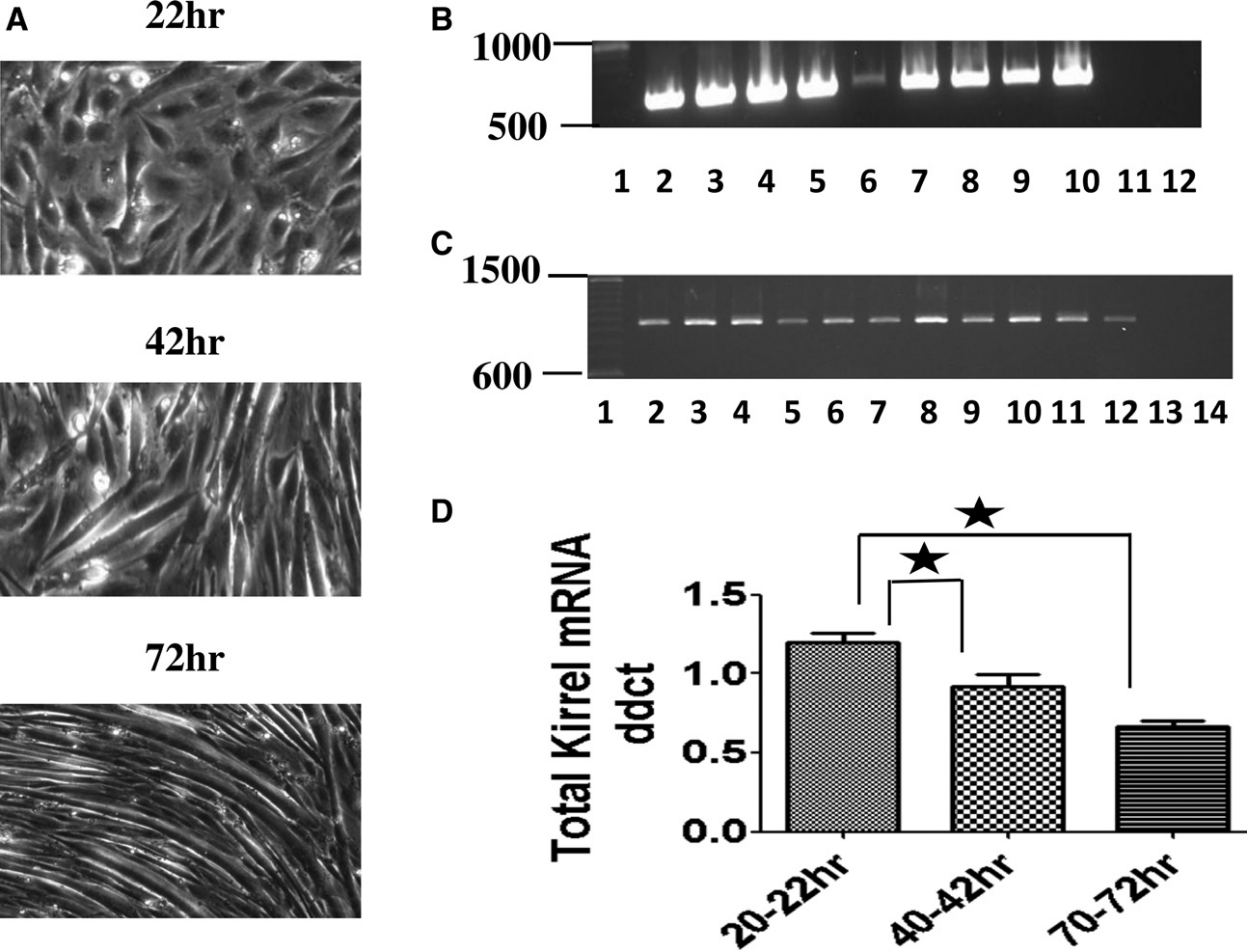
Expression of Kirrel mRNA transcripts during C2c12 differentiation and in additional rodent tissues. (A) Phase contrast images of C2C12 cells grown in DM for the indicated time durations. (B) PCR amplicons obtained from PCR using Kirrel A-specific primer set to detect Kirrel A. Lane 1 – 100 bp DNA ladder; Lanes 2–5 – C2C12 differentiation time course 0, 22, 42, and 72 h; Lane 6 – rat skeletal muscle; Lane 7 – mouse eye; Lane 8 – mouse brain; Lane 9 – mouse heart; Lane 10 – mouse kidney; Lane 11 – empty; and Lane 12 – negative control. (C) Amplicons obtained from PCR using Kirrel B-specific primer set to detect Kirrel B. (C) Lane 1 – 100 bp Ladder; Lanes 2–11 – C2C12 differentiation time-course samples 0, 20, 21, 22, 40, 41, 42, 70, 71, and 72 h; Lane 12 – mouse brain; Lane 13 – empty; and Lane 14 – negative control. (D) qPCR results for total Kirrel mRNA expression levels (i.e., Kirrel A and B combined) during C2C12 differentiation. 20–22 h (20, 21, and 22 h), 40–42 h (40, 41, and 42 h), 70–72 h (70, 71, and 72 h). Results are representative of *n* = 3 separate experiments. ⋆ Represents statistical significance obtained at *P* < 0.05.

To examine for the presence of both Kirrel A and B mRNA transcripts in C2C12 cells, a two-step RT-PCR strategy with transcript-specific primer sets was employed. Multiple time points were assessed during C2C12 differentiation, whereby C2C12 cells move from a mononucleated state to form multinucleated myotube like structures via somatic cell fusion events (Fig. 3A). Additional mouse and rat muscle tissues were also analyzed. The Kirrel A primer set had an expected amplicon of 555 nucleotides, whereas the Kirrel B primer set had an expected amplicon of 968 nucleotides. As a positive control for the Kirrel A primer set, murine brain and rat kidney tissues were included as Kirrel A has been previously reported to be present in these tissues (Gerke et al. 2006; Liu et al. 2003). Amplicons of the expected size were obtained for Kirrel A in all C2C12 differentiation time-course samples 0, 22, 42, and 72 h (Lanes 2–5 Fig. 3B) and in all mouse and rat tissues analyzed (Lanes 6–10 Fig. 3B), thus providing evidence that the Kirrel A transcript is present in C2C12 cells and also in adult rat skeletal muscle (Lane 6 Fig. 3B). Additional primer sets were used to confirm the presence of Kirrel A (data not shown). Amplicons of the expected size were also obtained for Kirrel B in all differentiation time-course samples from C2C12 cells (Lanes 2–11 Fig. 3C) and mouse brain (Lane 12 Figure 3C). Presence of a Kirrel B transcript in C2C12 cells was confirmed with two additional primer sets (data not shown). It was subsequently of interest to obtain quantitative mRNA expression data on Kirrel during C2C12 differentiation. Attempts were made to obtain quantitative expression data on the individual Kirrel splice variants; however, difficulties were encountered in obtaining consistent reliable expression data on Kirrel B as it appears to be expressed at a much lower level than Kirrel A. A focus was therefore put on analysis of total Kirrel mRNA levels via the use of a Taq Man probe which could detect both Kirrel A and Kirrel B mRNA transcripts. A gradual reduction in total Kirrel mRNA expression levels as differentiation progressed between Days 1 and 3 was observed (Fig. 3D). Statistically significant changes (mean + SEM) of ∼1.5- to 2-fold in relative Kirrel mRNA expression levels were found between 20 and 22 h versus 40 and 42 h (1.2 + 0.05 vs. 0.92 + 0.07; *P* < 0.05), 20 and 22 h versus 70 and 72 h (1.2 ± 0.05 vs. 0.66 ± 0.03; *P* < 0.05), and 40 and 42 h versus 70 and 72 h (0.92 ± 0.07 vs. 0.66 ± 0.03; *P* < 0.05).

### Expression profiling of Kirrel A and B protein Isoforms

**Figure 4:**
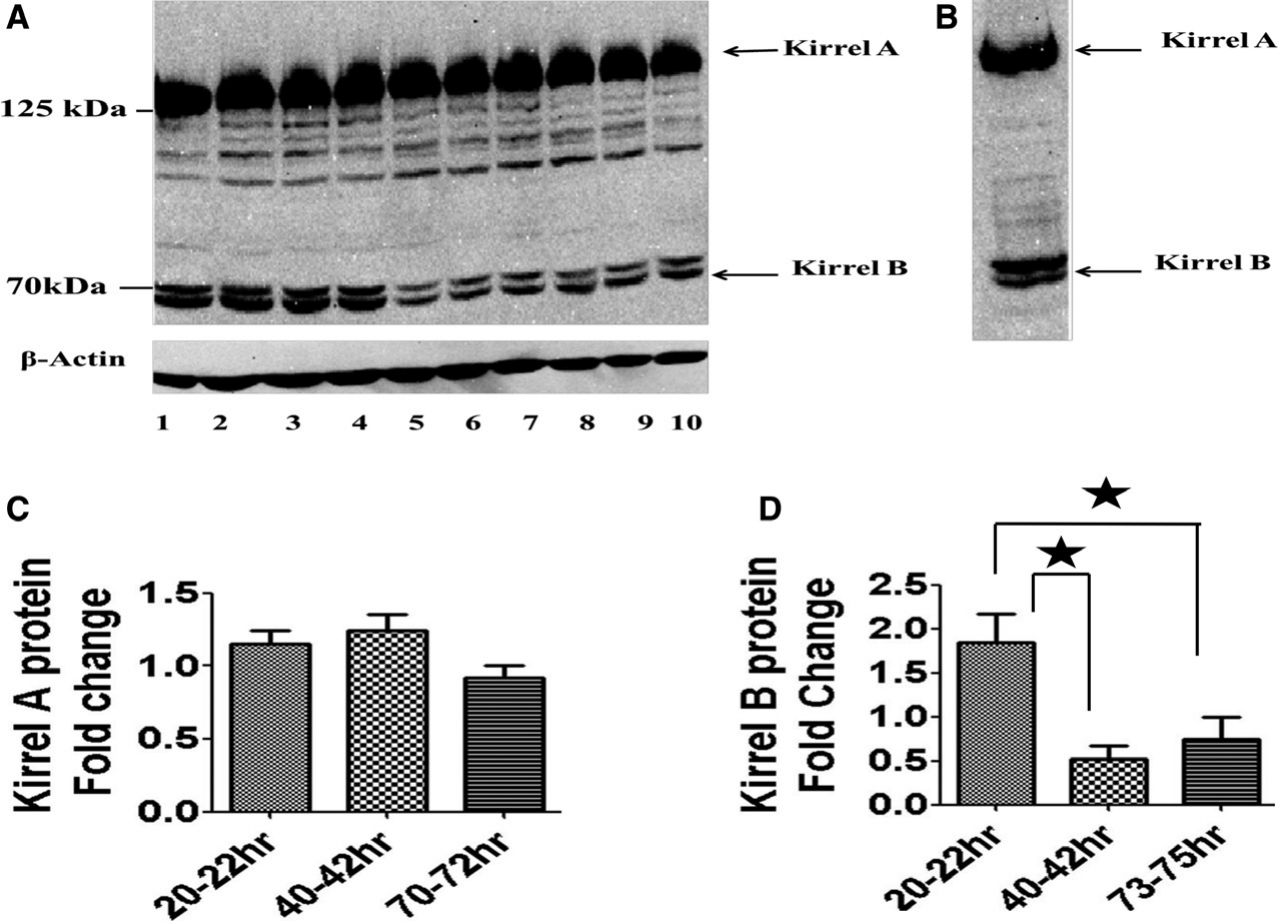
SDS-PAGE and immunodetection of Kirrel. (A) Lanes 1–10 – C2C12 differentiation time course 0, 20, 21, 22, 40, 41, 42, 70, 71, and 72 h. Results are representative of *n* = 3 separate experiments. (B) Mouse brain. (C) Relative protein expression levels of Kirrel A (i.e., ∼125 kDa immunoreactive band) during C2C12 differentiation time course. (D) Relative Kirrel B (i.e., ∼70 kDa band) protein expression during C2C12 differentiation time course. Results obtained from *n* = 3 separate experiments. ⋆ Represents statistical significance obtained at *P* < 0.05.

To assess for the possible protein presence of both Kirrel A and B in C2C12 cells during in vitro myogenesis, a commercial antibody was utilized. The predicted molecular weights of Kirrel A and B were 87.19 kDa and 69.98 kDa, respectively (http://www.bioinformatics.org/sms/prot_mw.html). Murine brain tissue was also included for analysis as Kirrel has previously been reported to be expressed in this tissue (Gerke et al. 2006) and our RT-PCR studies had suggested that Kirrel B was also present in the mouse brain. Multiple immunoreactive proteins between ∼90 and 125 kDa were detected in C2C12 cells (Fig. 4A Lanes 1–10) with the most intense signal present at ∼125 kDa, which is in close size agreement with previous reports of Kirrel detection in the mouse kidney (Liu et al. 2003). Additional strong and consistent immunoreactive proteins were also detected at ∼70 kDa which closely matches the predicted size of Kirrel B. Immunoreactive proteins at ∼125 and 70 kDa were also detected in murine brain (Fig. 4B) and hence were in agreement with our RT-PCR data. Prior incubation of the anti-Kirrel antibody with a blocking peptide resulted in either the total or almost complete elimination of immunoreactive proteins in C2C12 cells and mouse brain (data not shown), thus suggesting that these are Kirrel immunoreactive proteins. Quantitative analysis of the expression pattern of Kirrel A (125 kDa) and B (70 kDa – smallest of the two proteins) during C2C12 differentiation found no significant difference occurring in expression of Kirrel A (Fig. 4C). Statistically significant increased expression of approximately threefold and twofold was observed in Kirrel B protein levels at 20–22 h compared to 40–42 and 70–72 h, respectively (1.84 ± 0.32 vs. 0.51 ± 0.14 AU, *P* < 0.05; and 1.84 ± 0.32 vs. 0.74 ± 0.24 AU, *P* < 0.05) (Fig. 4D).

### Characterization of Kirrel mRNA and protein expression in response to chemical inhibition of the cell fusion process

**Figure 5:**
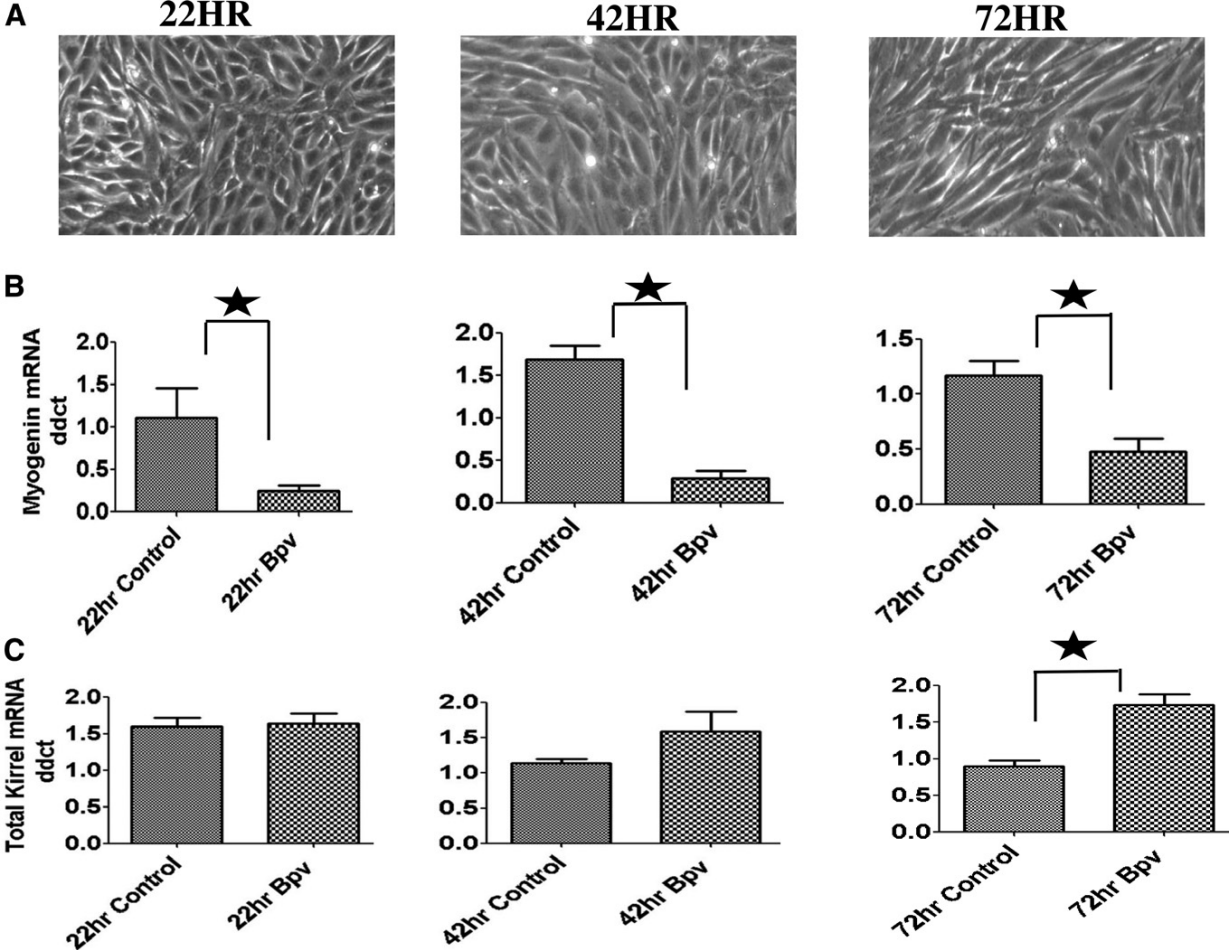
(A) Phase contrast images of C2C12 cells which had been treated with 10 μmol/L BpV. For comparison of difference in fusion inhibition to non-BpV–treated cells compare with Figure 2A. (B) Relative myogenin mRNA expression levels in control C2C12 cells and those treated with 10 μmol/L BpV during differentiation time course. (C) Relative Kirrel mRNA expression levels in control C2C12 cells and those treated with 10 μmol/L BpV during differentiation time course. ⋆ Represents statistical significance obtained at *P* < 0.05. Results are representative of *n* = 3 separate experiments.

To continue our investigation of Kirrel expression during in vitro myogenesis and to investigate a possible correlation with cell fusion, we utilized the BpV chemical compound which is a phosphotyrosine phosphatase inhibitor and has previously been demonstrated to significantly alter the cell fusion process in C2C12 cells (Castaldi et al. 2007), and is confirmed in our studies also (Fig. 5A). The impairment of the cell fusion process is associated with significant decreases in expression of the muscle-specific transcription factor myogenin. The expression of myogenin mRNA in BpV-treated cells compared to control cells (Fig. 5B) is significantly decreased by approximately fivefold (0.28 ± 0.07 vs. 1.68 ± 0.13; *P* < 0.01) after 42 h and approximately twofold (0.68 ± 0.16 vs. 1.16 ± 0.11; *P* < 0.05) 72 h postaddition of DM. At the 22-h time point, a clear trend toward lower expression in the BpV-treated samples compared to control was also observed (0.24 ± 0.05 vs. 1.1 ± 0.28; *P* = 0.06). In comparison to myogenin mRNA levels, no statistically significant difference was observed in total Kirrel mRNA levels (Fig. 5C) at 22 h or 42 h between control and BpV-treated cells; however, a significant increase in Kirrel mRNA of approximately twofold was observed in BpV-treated cells compared to control at 72 h (1.73 ± 0.11 vs. 0.89 ± 0.06; *P* < 0.05).

**Figure 6:**
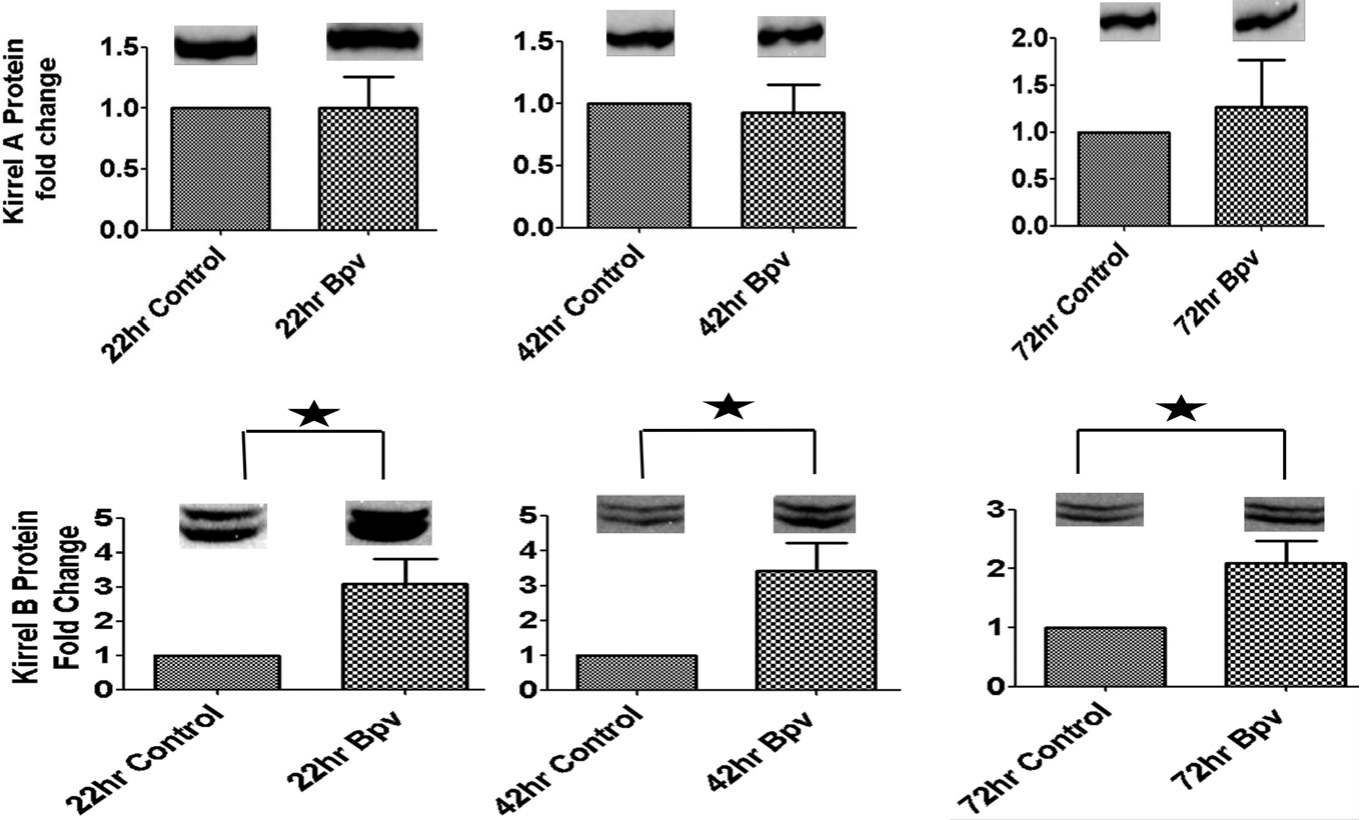
(A) Relative fold change of Kirrel A (∼125 kDa) protein expression levels in control and 10 μmol/L BpV-treated C2C12 cells at indicated time points during C2C12 differentiation time course. (B) Relative fold change in Kirrel B (∼70 kDa) protein expression levels in control and 10 μmol/L BpV-treated C2C12 cells at indicated time points during C2C12 differentiation time course. (C) B-Actin loading control. ⋆ Represents statistical significance obtained at *P* < 0.05. Results are representative of *n* = 3 separate experiments.

No statistically significant difference was observed in Kirrel A protein levels between BpV treated and control at any of the time points analyzed (Fig. 6A). In comparison at 22 and 42 h, Kirrel B expression levels were approximately threefold higher in BpV-treated cells compared to control (3.10 ± 0.72 and 3.43 ± 0.79, *P* < 0.05; Fig. 6B), whereas at 72 h, Kirrel B expression levels were approximately twofold (2.09 ± 0.38; *P* < 0.05 Fig. 6B) higher in BpV-treated cells compared to control.

## Discussion

Our understanding of the somatic cell fusion process in mammals which is a critical component for skeletal muscle development is currently at a rudimentary stage. Somatic cell fusion is recognized as being essential for numerous developmental and postnatal physiological processes including trophoblast development (Dupressoir et al. 2009), skeletal muscle growth (Horsley and Pavlath 2004), and osteoclast function (Ishii and Saeki 2008). Furthermore, evidence is also beginning to emerge that the occurrence of somatic cell fusion may be more widespread than previously recognized, as evidence of somatic cell fusion events have also been reported in brain (Alvarez-Dolado et al. 2003; Johansson et al. 2008), intestinal epithelium (Davies et al. 2009), liver (Faggioli et al. 2008), prostate (Placencio et al. 2010), and also cancer cells (Carter 2008; Duelli and Lazebnik 2003). Such findings are suggestive that improved mechanistic understanding of the somatic cell fusion process may elucidate novel therapeutic tools. Utilizing somatic cell fusion as a therapeutic aid has been attempted for treatment of muscular dystrophy in humans (Mendell et al. 1995; Miller et al. 1997). However, to date it has been unsuccessful in generating clinically beneficial results thus further supporting the need for increased knowledge of the molecular underpinnings of the somatic cell fusion process.

In light of such findings we investigated the Kirrel gene, a mammalian homolog of the *Drosophila* genes Rst and Kirre (Neumann-Haefelin et al. 2010), which have been shown to be key regulators of muscle cell fusion events during embryonic development in *Drosophila* (Strünkelnberg et al. 2001). Currently, rudimentary knowledge is available regarding Kirrel in mammalian skeletal muscle. Our results highlight the presence of two Kirrel transcripts in murine skeletal muscle cells and in the murine brain which is a previously unreported finding. The resultant proteins predicted to be encoded by these transcripts we term Kirrel A and Kirrel B. Significant structural differences are predicted to exist in the cytoplasmic domains of Kirrel A and B, which likely confer differential signaling capabilities to either isoform. Of particular note with regards to cell fusion is the loss of the GRB2-binding motif from Kirrel B. The GRB2-binding motif present in Kirrel A has been demonstrated to enable Kirrel A to induce actin nucleation at the plasma membrane of mammalian cells (Garg et al. 2007). Actin nucleation at the plasma membrane is essential for the muscle cell process in *Drosophila* and also in mammals (Abmayr and Pavlath 2012; Sens et al. 2010). Expression profiling of Kirrel B protein levels during in vitro myogenesis found its expression to be highest 1 day postaddition of DM, lowest 2 days postaddition of DM, and intermediate expression levels 3 days postaddition of DM. This expression pattern inversely matches the rate of occurrence of cell fusion events of C2C12 cells which is lowest 1 day postaddition of DM and highest 2 days postaddition of DM (Veliça and Bunce 2011). Chemical inhibition of the cell fusion process via treatment with BpV leads to significant increases in Kirrel B protein levels compared with controls; thus, mirroring the results obtained under standard differentiation culture conditions and further linking Kirrel B protein expression levels inversely with the occurrence of cell fusion events in skeletal muscle cells. Surprisingly we observed that Kirrel A protein expression levels, as assayed via the presence of a likely heavily posttranslationally modified immunoreactive band migrating at ∼125 kDa, did not display any significant perturbations in expression levels such as those described for Kirrel B. Our findings with regards to Kirrel A protein levels were similar to those observed for total Kirrel mRNA expression levels, which displayed small alterations during in vitro myogenesis and in response to our experimental interventions.

The structural analysis of Kirrel A and B highlights that both isoforms share an identical extracellular domain, therefore, Kirrel A and B may compete for interaction with the same extracellular ligand. The recent finding with regards to the importance of the murine homolog of SnS, Nephrin in murine skeletal muscle cell fusion, and the expression pattern of Nephrin being positively correlated with muscle cell fusion events (Sohn et al. 2009) makes it tempting to hypothesize that the decreased expression of Kirrel B when fusion rates are high may enable increased interactions between Kirrel A and Nephrin as protein expression levels of Kirrel A remain relatively constant throughout the differentiation time course of C2C12 cells. Such a scenario of Kirrel A and Nephrin interaction would mirror research findings from *Drosophila* Schneider cells demonstrating that Kirre and SnS can interact in trans (Galletta et al. 2004) and that they colocalize in trans in vivo at sites undergoing muscle cell fusion during embryonic development in Sens et al. (2010). Intriguingly in mouse L fibroblasts Kirrel A has been reported to interact in trans with Nephrin (Heikkilä et al. 2011), thus providing support for the possibility that a similar interaction may occur in mammalian muscle cells.

Notably in *Drosophila* at sites of fusion where SnS and Kirre colocalize in trans, significant actin nucleation occurs (Sens et al. 2010), likely due to the recruitment of pronucleation factors by Kirre and SnS (Abmayr and Pavlath 2012; Richardson et al. 2008). If components of the actin nucleation pathway such as the Wiskott Aldrich protein (WASP) are absent from muscle cells, the fusion process is blocked at the cell–cell adhesion stage between Kirre and SnS (Sens et al. 2010), thus highlighting the importance of localized actin nucleation at the cell membrane to the cell fusion process. Considering that Kirrel B lacks the GRB2-binding domains which enable Kirrel A to induce localized actin nucleation at the plasma membrane of mammalian cells (Garg et al. 2007), it could be hypothesized that Kirrel B may negatively regulate the cell fusion process by sequestering potential trans interactions of Kirrel A with other profusion partners such as Nephrin. Actin nucleation may therefore be prevented from occurring in the cell which expresses Kirrel B, thus, inhibiting cell fusion events. In *Drosophila*, it has been shown that actin nucleation occurs in both the cells which are undergoing fusion (Sens et al. 2010).

It is noteworthy that Kirrel B protein expression levels are inverse to the expression levels of myogenin, which is a key transcription factor for skeletal muscle differentiation (Rawls et al. 1995), a process which is characterized by the formation of multinucleated muscle fibers in vivo. Interestingly it has been demonstrated that as C2C12 cells differentiate and begin to form multinucleated myotubes, changes occur in the alternative splicing of mRNA transcripts (Bland et al. 2010). Therefore, the alternative splicing of Kirrel to yield both A and B protein isoforms may be indirectly regulated by myogenin via the ability of myogenin to drive the differentiation program of skeletal muscle cells and, hence, alter splicing events of mRNA transcripts such as Kirrel in skeletal muscle cells. Decreased production of the Kirrel B mRNA transcript due to changes in the spliceosome as a result of the differentiation process may as hypothesized above favor cell fusion events via enabling Kirrel A and Nephrin interaction. Overexpression studies of the Kirrel B isoform should provide an answer as to whether Kirrel B may be capable of inhibiting the somatic cell fusion process of skeletal muscle.

In the mammalian genome there are two additional Kirrel family members Kirrel2 and Kirrel3 (Neumann-Haefelin et al. 2010). Currently it remains unclear whether Kirrel3 is present or absent in mammalian skeletal muscle. A Kirrel3 mRNA transcript was not detected in an analysis of murine skeletal muscle (Ueno et al. 2003). However, contrary to this, it has been reported that sera raised against Kirrel3 detected a strong immunoreactive band in murine skeletal muscle at approximately 100 kDa which the authors suggest to possibly be a posttranslationally modified form of Kirrel3 (Gerke et al. 2005). Furthermore, it has been reported that in embryonic murine skeletal muscle (E17.5), Kirrel3 and Nephrin coimmunoprecipitate (Morikawa et al. 2007). This interaction was suggested as occurring at muscle spindle sites due to results from in situ hybridization studies, which found Kirrel3 to be present in proprioceptive neurons of the dorsal root ganglia while Nephrin was reported to be present in Neurotrophin 3–positive intrafusal muscle fibers. It will therefore be of interest to examine in detail the entire Kirrel family and possible interactions with Nephrin to ascertain their possible multiple diverse functions during myogenesis. Due to the highly similar extracellular domain present among mammalian Kirrel, Kirrel2, and Kirrel3 and their shared capability of interacting with similar proteins such as Podocin (Sellin et al. 2003), the possibility of redundancy among this family of genes is suggested. Such potential redundancy will need to be considered when attempting to elucidate how these genes may be involved in developmental processes, such as somatic cell fusion, where the absence of one family member, generated using knock-out technology, may be compensated for by another and hence no overt developmental or physiological defects may be displayed.

The presence of Kirrel B in murine brain tissue also requires further examination as the *C. elegans* homolog of Kirrel, SYG-1, is known to be involved in synaptogenesis (Wanner et al. 2011). Kirrel has been reported to be present at synapses in the murine brain (Gerke et al. 2006), however, the antibody used was directed toward the extracellular domain of Kirrel and so would not have been able to distinguish between Kirrel A and B. With the development of isoform-specific antibodies it will be of interest to examine the spatial distribution of both Kirrel isoforms in murine tissues. Finally, 3′ and 5′ race experiments will be required to examine whether both Kirrel isoforms originate from the same genomic locus and whether the untranslated regions of the transcripts vary between cell types. Such information will help elucidate possible regulatory mechanisms such as micro-RNAs (miRNAs) controlling expression of the alternatively spliced Kirrel transcripts in different cell types.
